# Xanthogranulomatous Cholecystitis: A Retrospective Review of Clinical Diagnosis and Treatment from a Single Center

**DOI:** 10.3390/healthcare12212184

**Published:** 2024-11-01

**Authors:** Mehmet Torun, Cebrail Akyüz, Deniz Kol, Mehmet Ali Özbay

**Affiliations:** 1 Gastrointestinal Surgery Clinic, Kosuyolu Yuksek Ihtisas Research and Training Hospital, University of Health Sciences, Istanbul 34865, Turkey; drcakyuz@hotmail.com; 2 General Surgery Clinic, Haydarpasa Numune Research and Training Hospital, University of Health Sciences, Istanbul 34865, Turkey; deniz.kol@sbu.edu.tr (D.K.); mehmetali.ozbay@sbu.edu.tr (M.A.Ö.)

**Keywords:** xanthogranulomatous cholecystitis, differential diagnosis, magnetic resonance imaging, computed tomography

## Abstract

The objective of this study was to evaluate and compare the histopathological, clinical, and treatment characteristics of xanthogranulomatous cholecystitis (XGC) in patients undergoing cholecystectomy at a single center. **Aim**: We aim to enhance the understanding of its presentation and improve its differential diagnosis from other gallbladder pathologies. **Methods**: We retrospectively reviewed 6783 cholecystectomy cases performed between January 2015 and January 2023 at the General Surgery Clinic of Haydarpaşa Numune Training and Research Hospital, and a diagnosis of xanthogranulomatous cholecystitis was histopathologically established in 131 patients. In this retrospective study, we examined the clinicopathological characteristics, preoperative imaging methods and findings, histopathological images, surgical procedure methods, and postoperative complications of 131 patients. **Results**: The study included 131 patients, with ages ranging from 18 to 88 years, of which 74 (56.5%) were female and 57 (43.5%) were male. Ultrasound imaging was performed on 128 patients. Ultrasound imaging revealed wall thickening in 72.7% of cases, hypoechoic nodules in 13.3%, biliary tract pathologies in 10.9%, and adenomyomatosis in 3.1%. A total of 59 cases had MRI. On MRI, wall thickening was observed in 50.8% of cases, biliary tract pathologies in 33.9%, adenomyomatosis in 10.2%, hypoechoic nodules in 3.4%, and hypoechoic nodules + wall thickening (HN + WT) in 1.7%. Histopathological diagnosis was diffuse in 79.4% of cases and focal in 20.6%. In addition to cholecystectomy, non-surgical interventions were not required in 77.1% of the cases, while 11.5% underwent ERCP, 9.2% underwent percutaneous procedures, 1.5% underwent both ERCP and percutaneous procedures, and 0.8% underwent other non-surgical interventions. Of the surgeries, 93.1% were elective and 6.9% were emergency. Postoperative complications were not observed in 84% of the patients; 5.3% experienced surgical complications, 5.3% had surgical site infection, and 5.3% had other complications (pneumonia and urinary infection). The length of hospital stay ranged from 0 to 26 days, with a mean of 5.27 ± 4.59 days and a median of 4 days. **Conclusions**: Xanthogranulomatous cholecystitis is a rare disease of the gallbladder with no characteristic radiological or clinical findings and can often be confused with gallbladder cancer. Further studies involving larger populations are needed to improve the preoperative diagnosis.

## 1. Introduction

Xanthogranulomatous cholecystitis (XGC) is a rare inflammatory disease of the gallbladder (GB). Inflammation can lead to asymmetric wall thickening and nodule formation in the gallbladder wall [1]. Despite being benign, its macroscopic appearance can mimic that of gallbladder cancer [2]. Clinically, XGC can present as acute or chronic cholecystitis. Differential diagnosis between XGC and gallbladder cancer is particularly challenging in patients with severe fibrosis of the gallbladder and the surrounding organs [3]. Preoperative imaging methods such as ultrasonography (USG), magnetic resonance imaging (MRI), and computed tomography (CT) may reveal similar findings in XGC and acute cholecystitis, and intraoperative frozen section analysis may be necessary to exclude the diagnosis of gallbladder cancer in selected cases. An effective preoperative diagnosis in these patients appears to be crucial; however, a definitive diagnosis can only be made histopathologically [4]. This study aimed to analyze 131 cases of XGC that were histopathologically confirmed after cholecystectomy over a 10-year period. The objective of this study was to evaluate the clinical and radiological presentations, surgical approaches, and postoperative complications of XGC cases and to compare these findings with the existing literature to enhance the understanding of its diagnosis and management.

## 2. Material and Methods

This retrospective study was conducted at the General Surgery Clinic of Haydarpaşa Numune Training and Research Hospital. A total of 6783 patients who underwent cholecystectomy between January 2015 and January 2023 were reviewed. Among these, 131 were histopathologically diagnosed with XGC. The retrospective nature of this study was designed to capture detailed clinical presentations, imaging findings, and surgical outcomes to enhance the understanding of XGC, without a direct comparative analysis of other gallbladder pathologies.

### 2.1. Study Design and Patient Selection

Patients who underwent cholecystectomy and were confirmed to have XGC through a histopathological examination were included in the study. Patients with other gallbladder pathologies or with incomplete medical records were excluded from the analysis. The inclusion criteria were a histopathological confirmation of XGC, while the exclusion criteria included cases without definitive pathology reports or incomplete clinical data. Due to the descriptive nature of the study, which focused on XGC, a control group was not included. While this study focused on the clinicopathological characteristics of XGC, we acknowledge that the lack of a control group limits direct comparisons with gallbladder cancer or other cholecystitis types. The absence of a control group in this study was because of the specific aim of providing a focused analysis of the unique clinical and histopathological characteristics of XGC.

Data Collection: data were extracted from medical records, including patient demographics (age and sex), clinical presentations (symptoms, duration), preoperative imaging (ultrasonography, computed tomography (CT), magnetic resonance imaging (MRI)), surgical procedure details (laparoscopic or open cholecystectomy), and postoperative outcomes (complications and length of hospital stay).

Histopathological Examination: Histopathological analysis was performed by two experienced pathologists specializing in hepatobiliary disease. The diagnosis of XGC was established on the basis of the presence of characteristic histological features, including xanthoma cells, chronic inflammation, and fibrosis. All specimens underwent standard histopathological processing, and the final diagnosis was confirmed based on a consensus between the two pathologists.

Radiological Assessment: Preoperative imaging data were analyzed to evaluate the radiological characteristics of XGC. Ultrasound (USG), CT, and MRI were performed by radiologists with expertise in abdominal imaging, and findings, such as gallbladder wall thickening, the presence of hypoechoic nodules, and biliary tract abnormalities, were documented. The criteria for radiological diagnosis were based on the standard imaging protocols for gallbladder pathology.

Surgical Approach and Intraoperative Findings: The type of surgery (laparoscopic or open cholecystectomy) was determined based on the preoperative imaging and intraoperative findings. Conversion from laparoscopic to open surgery was also recorded. Intraoperative frozen section analysis was performed in cases where malignancy was suspected to confirm the diagnosis and guide surgical decision making.

Postoperative Follow-Up: Patients were followed-up postoperatively to monitor for any complications, including surgical site infection, bile leakage, and other morbidities. The length of hospital stay and readmission within 30 days of surgery were documented.

### 2.2. Ethics and Statistical Analysis

This study was approved by the Ethics Committee of the Governorship Provincial Health Directorate Haydarpaşa Numune Training and Research Hospital (approval number: E-62977267-771-238832024, date: 27 February 2024). The requirement for informed consent was waived, owing to the retrospective nature of the study.

### 2.3. Statistical Analysis

Statistical analyses were performed using IBM SPSS Statistics version 22. Continuous variables were summarized using descriptive statistics, including mean, standard deviation (SD), and median, minimum, and maximum values. For normally distributed data, means and standard deviations were reported, whereas for non-normally distributed data, medians with interquartile ranges were provided. Categorical variables are expressed as frequencies and percentages. The chi-squared test was used to compare categorical data. Fisher’s exact chi-squared test was applied to ensure accurate results when the expected frequency was <5 in any cell. For comparisons involving variables with more than two categories or for multi-way tables, the Fisher–Freeman–Halton exact chi-squared test was used to assess the association between the variables. For all statistical analyses, a *p*-value of less than 0.05 was considered to indicate statistical significance.

## 3. Results

A total of 131 patients, ranging in age from 18 to 88 years, were included in the study, of whom 74 (56.5%) were female and 57 (43.5%) were male. The mean age was 55.81 ± 13.91 years. The demographic characteristics of patients are presented in Table 1.

Ultrasonography (USG) was performed in 128 patients who underwent USG imaging. Among them, 72.7% showed wall thickening, 13.3% had hypoechoic nodules, 10.9% exhibited biliary tract pathologies, and 3.1% had adenomyomatosis. Additionally, 76 patients underwent CT imaging, of whom 76.3% displayed wall thickening, 21.1% showed biliary tract pathologies, and 2.6% had adenomyomatosis. Furthermore, 59 patients underwent MRI, with 50.8% showing wall thickening, 33.9% exhibiting biliary tract pathologies, 10.2% adenomyomatosis, 3.4% hypoechoic nodules, and 1.7% hypoechoic nodules combined with wall thickening. Finally, 68 patients underwent magnetic resonance cholangiopancreatography (MRCP), with 51.5% displaying wall thickening, 36.8% exhibiting biliary tract pathologies, 8.8% showing adenomyomatosis, 1.5% presenting hypoechoic nodules, and 1.5% showing HN + WT (Table 2).

**Figure 1:**
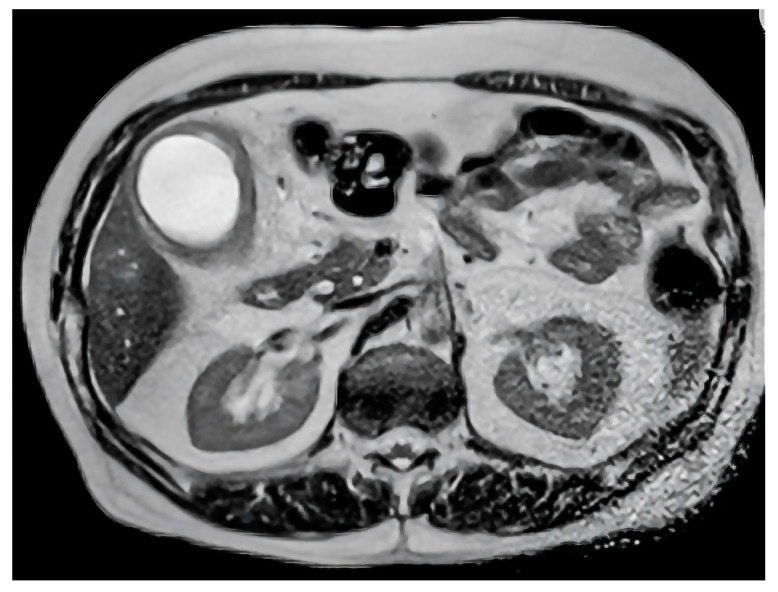
Axial T2-weighted MRI scan demonstrating gallbladder wall thickening and intramural nodules, characteristic of xanthogranulomatous cholecystitis.

**Figure 2:**
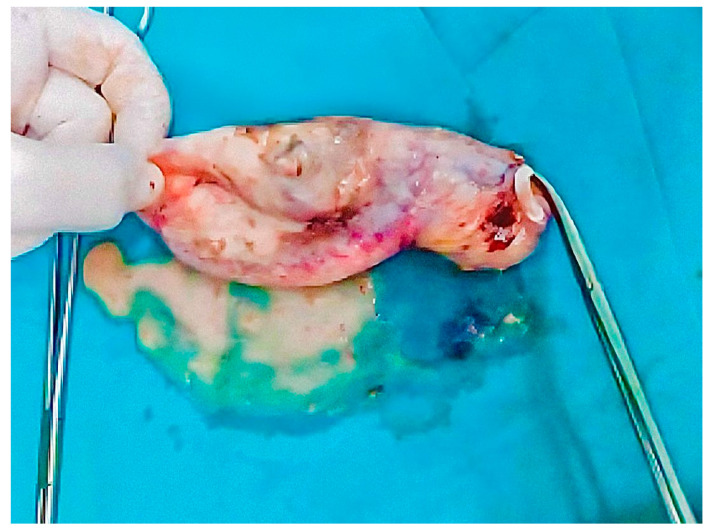
Gross appearance of the gallbladder specimen showing thickened walls and nodular appearance indicative of xanthogranulomatous cholecystitis.

**Figure 3:**
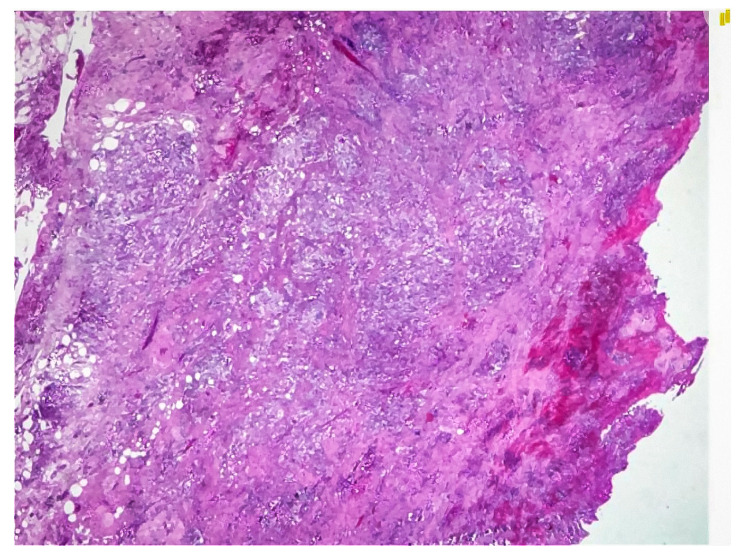
Hematoxylin and eosin (H&E) stained sections showing dense inflammatory infiltrates, xanthoma cells, and fibrosis consistent with xanthogranulomatous cholecystitis.

Intraoperative frozen section analysis was performed in 21.4% of cases. Laparoscopic surgery was performed in 49.6% of cases, open surgery in 15.3%, and conversion from laparoscopy to open surgery in 35.1%. Histopathologically, 79.4% of the cases had a diffuse diagnosis, whereas 20.6% had a focal diagnosis. Nonsurgical interventions were performed in 77.1% of cases, endoscopic retrograde cholangiopancreatography (ERCP) in 11.5%, percutaneous interventions in 9.2%, both ERCP and percutaneous interventions in 1.5%, and other nonsurgical interventions in 0.8% of cases. Of the operations, 93.1% were elective and 6.9% were emergency procedures. Postoperative complications were observed in 5.3% of the cases for both surgical complications and surgical site infections, as well as other complications (pneumonia, urinary infection, etc.). The length of hospital stay ranged from 0 to 26 days, with a mean of 5.27 ± 4.59 days and a median of 4 days (Table 3) (Figure 1, Figure 2 and Figure 3).

CA 19-9 levels were elevated (>35) in 19.3% of the patients (Table 4).

There was no statistically significant difference in the histopathological results based on the USG findings (*p* = 0.21). Among the cases with hypoechoic nodules, 82.4% had a diffuse histopathological diagnosis, while 80.6% of cases with wall thickening and 78.6% with biliary tract pathologies also had diffuse diagnoses. Similarly, no significant difference was found in the histopathological results based on CT findings (*p* = 0.32), with 81% of cases with wall thickening and 68.8% with biliary tract pathologies, and all cases with adenomyomatosis had a diffuse histopathological diagnosis. The same trend was observed for MRI findings (*p* = 0.45), with all cases of hypoechoic nodules, 76.7% of cases with wall thickening, 100% of cases with hypoechoic nodules combined with wall thickening, 75% of cases with biliary tract pathologies, and 83.3% of cases with adenomyomatosis with a diffuse histopathological diagnosis. Similarly, no statistically significant difference was observed in the histopathological results based on the MRCP findings (*p* = 0.38), with all cases of hypoechoic nodules, 80% of cases with wall thickening, 100% of cases with hypoechoic nodules combined with wall thickening, 76% of cases with biliary tract pathologies, and 83.3% of cases with adenomyomatosis with a diffuse histopathological diagnosis.

There was no statistically significant relationship between USG findings and surgical procedures (*p* = 0.25). Laparoscopic surgery was performed in 29.4% of cases with hypoechoic nodules, 51.6% with wall thickening, 57.1% with biliary tract pathologies, and 50% with adenomyomatosis. Similarly, there was no statistically significant relationship between CT findings and surgical procedures (*p* = 0.18), with laparoscopic surgery performed in 55.2% of the cases with wall thickening and 68.8% of the cases with biliary tract pathologies. For cases of adenomyomatosis, one patient underwent open surgery, while the other underwent conversion to open surgery. There was also no statistically significant relationship between MRI findings and surgical procedures (*p* = 0.30), and laparoscopic surgery was performed in 36.7% of cases with wall thickening, 60% of cases with biliary tract pathologies, and one case with hypoechoic nodules combined with wall thickening. Similarly, there was no statistically significant relationship between the MRCP findings and surgical procedures (*p* = 0.22) (Table 5).

Laparoscopic surgery was performed in 37.1% of cases with wall thickening, 56% of cases with biliary tract pathologies, and one case with hypoechoic nodules combined with wall thickening. All patients with hypoechoic nodules, and 50% of those with adenomyomatosis, underwent conversion to open surgery. There was no statistically significant relationship between histopathological diagnosis and surgical procedures (*p* > 0.05), and laparoscopic surgery was performed in 51.9% of cases with a focal diagnosis and in 49% of cases with a diffuse diagnosis (Table 6).

## 4. Discussion

Xanthogranulomatous cholecystitis is a rarely encountered disease defined as an inflammatory disorder [4]. Fibroxanthogranulomatous cholecystitis was first described by Christensen and Ishak (1970) [5] and later termed xanthogranulomatous cholecystitis by McCoy et al. (1976) [6]. The age of onset of the disease is generally reported to be 53 (49–62) in the literature [6]. In this study, the mean age of onset is 55.8 years, which is consistent with previous reports indicating a mean age of approximately 53 years, typically presenting in the fifth and sixth decades of life [6]. This study did not find a significant difference between male and female patients, which was consistent with some studies suggesting an equal distribution between the sexes, although others have reported a slight female predominance [7].

Gallstone disease and XGC co-existence are common [8]. Similar to other studies, we observed that all patients with XGC had cholelithiasis. The clinical presentations of XGC are diverse, with cholecystitis (77.1%) being the most common reason for admission, followed by cholangitis (18.3%) and pancreatitis (2.3%). These findings are consistent with earlier studies in which XGC often presented as acute or chronic cholecystitis, making its differentiation from gallbladder cancer challenging.

Since XGC does not have specific symptoms, it often presents as acute or chronic cholecystitis [5]. Owing to the possibility of confusion with gallbladder cancer, differential diagnosis can be challenging [9]. Zaheer et al. [10] reported that patients had experienced at least one episode of acute cholecystitis, and in six patients, this was the sole presenting symptom. A larger series of studies has also shown a frequent association between XGC diagnosis and patients diagnosed with acute cholecystitis [11]. Consistent with the literature, our study found that the patients had various clinical presentations, with cholecystitis and cholangitis being the most common reasons for admission. The patients presented with cholecystitis (77.1%), cholangitis (18.3%), and pancreatitis (2.3%).

If gallbladder cancer is suspected in the preoperative and differential diagnoses, radical cholecystectomy is indicated [12]. However, physical examination, laboratory results, and radiological imaging may not be helpful in differential diagnosis or ruling out cancer diagnosis. Gallbladder stones or focal or diffuse wall thickening can be detected by USG [13]. The presence of hypoechoic nodules is considered a characteristic finding, although these findings can also be observed in adenomyomatosis and intramural abscesses, making differentiation challenging. The role of imaging in the diagnosis of XGC remains controversial. Zaheer et al. found that a significant number of patients experienced at least one episode of acute cholecystitis, which was often the sole presenting symptom. In our study, imaging techniques such as ultrasonography (USG), computed tomography (CT), and magnetic resonance imaging (MRI) were used, but none were definitive for the preoperative diagnosis of XGC [13,14]. Although hypoechoic nodules on USG are considered characteristic, they can also be observed in conditions such as adenomyomatosis or abscesses, leading to diagnostic challenges. Wall thickening was a common finding across USG, CT, and MRI but did not aid in differentiating between focal and diffuse diseases. The literature reports that CT findings can sometimes assist in differentiating XGC from gallbladder carcinoma by showing features such as diffuse wall thickening, mucosal irregularity, and intramural nodules [15,16]. However, in our study, MRI was more sensitive in highlighting inflammatory cells and necrotic areas but still did not achieve a high correlation with histopathological findings [17,18].

The most distinctive feature of contrast-enhanced abdominal CT is an intramural nodule with a hypodense appearance [14]. Furthermore, CT criteria have been defined for the differential diagnosis of XGC and gallbladder diseases, and the presence of at least three criteria, including diffuse wall thickening, mucosal irregularity, the presence of intramural nodules, the absence of invasion to the liver parenchyma, and the absence of dilation in intrahepatic bile ducts, have been reported to have a sensitivity and specificity of 83% and 100%, respectively [15,16]. Several studies have compared CT and MRI findings, but MRI can reflect areas with low involvement, such as inflammatory cells and necrotic abscesses, in patients pathologically diagnosed with XGC, indicating that a high correlation cannot always be achieved [17]. In our study, although wall thickening was high on USG, MRI, and CT, it was not useful for the preoperative diagnosis of XGC, either focally or diffusely.

Tumor markers such as CA 19-9 and CEA are often elevated in patients with gallbladder cancer. In our study, CA 19-9 levels were elevated (>35) in 19.3% of patients, which is considerably lower than the sensitivity and specificity reported in previous studies [18,19]. This discrepancy underscores the variability in CA 19-9 elevation in XGC and its limited utility in distinguishing it from malignancy. The surgical management of XGC involves cholecystectomy, which is the definitive treatment. In line with other reports, our study found that most patients underwent laparoscopic surgery (49.6%), with some requiring conversion to open surgery (35.1%) because of dense adhesions and fibrotic changes characteristic of XGC. This conversion rate is higher than that observed in acute cholecystitis cases, as noted in other studies.

In cases diagnosed as XGC, the curative treatment is surgery, and only cholecystectomy seems sufficient [20]. However, XGC, due to fibrotic adhesions in the gallbladder and adjacent organs, fistula formation, the disruption of gallbladder integrity, and not mimicking the appearance of gallbladder cancer, can affect surgical procedures by preventing dissection [2]. In cases where differentiation between malignancy and XGC is difficult, intraoperative frozen section analysis can be useful [21]. Therefore, intraoperative frozen sections were performed in 21.4% of the patients with suspected XGC, preventing unnecessary extensive resection. If feasible initially, laparoscopic cholecystectomy can be performed in XGC cases; however, cases not conducive to laparoscopy or lacking dissection plans transition to open surgery, which is generally higher in XGC-diagnosed patients than in acute cholecystitis cases [22]. In our study, 49.6% of the patients underwent laparoscopic surgery, 15.3% underwent open surgery, and 35.1% underwent conversion from laparoscopy to open surgery. While one of the patients diagnosed with adenomyomatosis underwent open surgery, the other patient underwent conversion from laparoscopic to open surgery.

There was no statistically significant relationship between the MRI findings and surgical procedures. Among patients with wall thickening, 36.7% underwent laparoscopic surgery, while 60% of those with bile duct pathology and one with HN + WT underwent laparoscopic surgery. Two patients with hypoechoic nodules (100%) and 50% of those diagnosed with adenomyomatosis underwent conversion from laparoscopic to open surgery.

There was no statistically significant relationship between MRCP findings and surgical procedures. Among the patients with wall thickening, 37.1% underwent laparoscopic surgery, whereas 56% of those with bile duct pathology and one with HN + WT underwent laparoscopic surgery. One patient with hypoechoic nodules and 50% of those diagnosed with adenomyomatosis underwent conversion from a laparoscopic to open procedure.

There was no statistically significant relationship between histopathological diagnosis and surgical procedures. Laparoscopic surgery was performed in 51.9% of the cases with a focal diagnosis and in 49% of the cases with a diffuse diagnosis. This distribution is consistent with that reported in the literature [8,11,13]. Additionally, minor or major postoperative complications such as bile leakage, surgical site infection, and bleeding may occur in patients [4,23]. In our series, postoperative complications were not observed in 84% of the patients, while surgical complications were detected in 5.3% and surgical site infections in 5.3%. The length of hospital stay ranged from 0 to 26 days, with a mean of 5.27 ± 4.59 days and a median of 4 days.

### 4.1. Limitations of the Study

The primary limitation of our study was its retrospective design, which may introduce selection bias and limit the ability to establish causal relationships. In addition, the study was conducted at a single center, which may affect the generalizability of the results to other populations. The absence of a control group also limits the ability to directly compare the characteristics of XGC with those of other gallbladder pathologies. Furthermore, the small sample size of patients with rare presentations or findings may have influenced the statistical power to detect any significant differences. Finally, owing to incomplete medical records in some cases, certain clinical or radiological details might not have been thoroughly captured.

### 4.2. Implications for Future Clinical Practice and Strengths of the Study

This study provides valuable insights into the clinical and histopathological features of XGC, highlighting the challenges in preoperatively differentiating XGC from gallbladder malignancies. A comprehensive analysis of the imaging findings and their association with surgical outcomes offers important considerations for surgeons in preoperative planning and intraoperative decision making. One of the strengths of our study was the detailed evaluation of a relatively large number of XGC cases from a single center over a 10-year period, contributing to the literature on this rare condition. These findings may aid in improving diagnostic accuracy and optimizing management strategies for patients.

## 5. Conclusions

Xanthogranulomatous cholecystitis is a rare inflammatory disease of the gallbladder similar to gallbladder cancer. Our findings contribute to the growing understanding of the clinicopathological characteristics of XGC and offer insights into its presentation, diagnosis, and surgical management. Further studies with larger populations and control groups are required to improve the preoperative differentiation of XGC from other gallbladder pathologies.

## Figures and Tables

**Table 1:** Demographic characteristics and clinical presentation.

		n	%
Sex	Female	74	56.5
	Male	57	43.5
Clinical presentation	Cholecystitis	101	77.1
	Cholangitis	24	18.3
	Pancreatitis	3	2.3
	Other	3	2.3
Age		18–88	55.81 ± 13.91

**Table 2:** Radiological imaging findings.

Findings	USG (n = 128)		CT (n = 76)		MRI (n = 59)		MRCP (n = 68)	
	n	%	n	%	n	%	n	%
Hypoechoic nodule	17	13.3	0	0	2	3.4	1	1.5
Wall thickening	93	72.7	58	76.3	30	50.8	35	51.5
Biliary tract pathologies	14	10.9	16	21.1	20	33.9	25	36.8
Adenomyomatosis	4	3.1	2	2.6	6	10.2	6	8.8
HN + WT	0	0	0	0	1	1.7	1	1.5

USG: ultrasound CT: computed tomography MRI: magnetic resonance imaging; MRCP: magnetic resonance cholangio pancretografi; HN + WT: hypoechoic nodule + wall thickening.

**Table 3:** Descriptive characteristics of surgery, postoperative complications, and pathology results.

		n	%
Intraoperative frozen (n = 131)	No	103	78.6
	Yes	28	21.4
Histopathological diagnosis (n = 131)	Focal	27	20.6
	Diffuse	104	79.4
Surgical procedure (n = 131)	Laparoscopy	65	49.6
	Laparotomy	20	15.3
	Conversion Laparotomy	46	35.1
Postoperative complications (n = 131)	No Complication	110	84
	Surgical Complication	7	5.3
	Surgical Site	7	5.3
	Others	7	5.3
Non-surgical interventions (n = 131)	No	101	77.1
	Percutaneous	12	9.2
	1.2	2	1.5
	ERCP	15	11.5
	Others	1	0.8
Emergency–elective (n = 131)	Emergency	9	6.9
	Elective	122	93.1
Duration of hospital stay min–max, mean ± SD (median)		0–26	5.27 ± 4.59 (4)

**Table 4:** Descriptive features of Ca19-9 level.

		n	%
Ca 19-9 (n = 88)	>35	17	19.3
	<35	71	80.7
Ca 19-9_Min–Max, Ort±SS (median)_		1–47,894	654.7 ± 5109.9 (13)

**Table 5:** Relationship between radiological imaging and histopathological results.

		Histopathological Diagnosis		*p*
		Focal	Diffuse	
		n (%)	n (%)	
USG	Hypoechoic nodule	3 (%17.6)	14 (%82.4)	0.503
	Wall thickening	18 (%19.4)	75 (%80.6)	
	Biliary tract pathologies	3 (%21.4)	11 (%78.6)	
	Adenomyomatozis	2 (%50)	2 (%50)	
CT	Wall thickening	11 (%19)	47 (%81)	0.482
	Biliary tract pathologies	5 (%31.3)	11 (%68.8)	
	Adenomyomatozis	0 (%0)	2 (%100)	
MRI	Hypoechoic nodule	0 (%0)	2 (%100)	1.000
	Wall thickening	7 (%23.3)	23 (%76.7)	
	HN + WT	0 (%0)	1 (%100)	
	Biliary tract pathologies	5 (%25)	15 (%75)	
	Adenomyomatozis	1 (%16.7)	5 (%83.3)	
MRCP	Hypoechoic nodule	0 (%0)	1 (%100)	0.939
	Wall thickening	7 (%20)	28 (%80)	
	HN + WT	0 (%0)	1 (%100)	
	Biliary tract pathologies	6 (%24)	19 (%76)	
	Adenomyomatozis	1 (%16.7)	5 (%83.3)	

USG: ultrasound CT: computed tomography MRI: magnetic resonance imaging; MRCP: magnetic resonance colanvio pancretografi; HN + WT: hypoechoic nodule + wall thickness.

**Table 6:** Relationship between radiological imaging findings, histopathological results, and surgical procedures.

		Surgical Procedure			*p*
		Laparoscopy	Conventional	Conversion Laparotomy	
		n (%)	n (%)	n (%)	
USG	Hipoechoic nodül	5 (%29.4)	4 (%23.5)	8 (%47.1)	0.663
	Wall thickening	48 (%51.6)	14 (%15.1)	31 (%33.3)	
	Biliary tract pathologies	8 (%57.1)	1 (%7.1)	5 (%35.7)	
	Adenomyomatosis	2 (%50)	1 (%25)	1 (%25)	
CT	Wall thickening	32 (%55.2)	11 (%19)	15 (%25.9)	0.257
	Biliary tract pathologies	11 (%68.8)	1 (%6.3)	4 (%25)	
	Adenomyomatosis	0 (%0)	1 (%50)	1 (%50)	
MRI	Hypoechoic nodule	0 (%0)	0 (%0)	2 (%100)	0.156
	Wall thickening	11 (%36.7)	11 (%36.7)	8 (%26.7)	
	HN + WT	1 (%100)	0 (%0)	0 (%0)	
	Biliary tract pathologies	12 (%60)	2 (%10)	6 (%30)	
	Adenomyomatosis	2 (%33.3)	1 (%16.7)	3 (%50)	
MRCP	Hypoechoic nodule	0 (%0)	0 (%0)	1 (%100)	0.460
	Wall thickening	13 (%37.1)	11 (%31.4)	11 (%31.4)	
	HN + WT	1 (%100)	0 (%0)	0 (%0)	
	Biliary tract pathologies	14 (%56)	3 (%12)	8 (%32)	
	Adenomyomatosis	2 (%33.3)	1 (%16.7)	3 (%50)	
Histopathological Diagnosis	Focal	14 (%51.9)	5 (%18.5)	8 (%29.6)	0.755
	Diffuse	51 (%49)	15 (%14.4)	38 (%36.5)	

USG: ultrasound CT: computed tomography MRI: magnetic resonance imaging; MRCP: magnetic resonance colanvio pancretografi; HN + WT: hypoechoic nodule + wall thickness.

## Data Availability

The datasets generated and/or analyzed during the current study are available from the corresponding author upon reasonable request.

## Abbreviations

XGC	xanthogranulomatous cholecystitis
GB	gallbladder
USG	ultrasonography
CT	computed tomography
MRI	magnetic resonance imaging
MRCP	magnetic resonance cholangiopancreatography
ERCP	endoscopic retrograde cholangiopancreatography
CA 19-9	carbohydrate antigen 19-9
CEA	carcinoembryonic antigen
HN + WT	hypoechoic nodule and wall thickening
SD	standard deviation
